# Prevalence and Correlates of Prenatal Vitamin A Deficiency in Rural Sidama, Southern Ethiopia

**DOI:** 10.3329/jhpn.v31i2.16382

**Published:** 2013-06

**Authors:** Samson Gebremedhin Gebreselassie, Fikre Enquselassie Gase, Melaku Umeta Deressa

**Affiliations:** ^1^School of Public and Environmental Health, Hawassa University, PO Box 12485, Addis Ababa, Ethiopia;; ^2^School of Public Health,; ^3^School of Medicine, Addis Ababa University, Ethiopia

**Keywords:** Maternal nutrition, Nutritional surveys, Serum retinol, Vitamin A deficiency, Ethiopia

## Abstract

A cross-sectional study was conducted to assess the prevalence and correlates of prenatal vitamin A deficiency (VAD) in rural Sidama, Southern Ethiopia. Seven hundred randomly-selected pregnant women took part in the study. Serum retinol concentration was determined using high-performance liquid chromatography. Data were analyzed by logistic and linear regression. Interpretation of data was made using adjusted odds ratio (AOR) and adjusted linear regression coefficient. The prevalence of VAD (serum retinol <0.7 µmol/L) was 37.9%. Advanced gestational age and elevated C-reactive protein (CRP ≥5 mg/dL) were negatively associated with retinol concentration (p<0.05). The odds of VAD was significantly higher among the women with no education and those devoid of self-income. Women aged 35-49 years had 2.23 (95% CI 1.31-3.81) times higher odds compared to those aged 15-24 years. The lower the dietary diversity score in the preceding day of the survey, the higher were the odds of VAD. With reference to nulliparas, grand multiparas had 1.92 (95% CI 1.02-3.64) times increased odds of VAD. VAD and zinc deficiency (serum zinc <8.6 µmol/L during the first trimester, or <7.6 µmol/L during the second or third trimester) were significantly associated with AOR of 1.80 (95% CI 1.28-2.53). VAD has major public-health significance in the area. Accordingly, it should be combated through enhancement of diet diversity, birth control, and socioeconomic empowerment of women.

## INTRODUCTION

Vitamin A is an essential micronutrient required for the normal functioning of the vision system, immunity, epithelial integrity, cellular differentiation, growth and development, and possibly reproduction (1,2). The World Health Organization (WHO) defines vitamin A deficiency (VAD) as tissue concentrations of vitamin A (VA) low enough to have adverse health consequences, even if there is no evidence of clinical deficiency (3).

VAD remains a serious public-health problem in the developing world (4). Preschool children and pregnant women suffer the most widespread and severe effects of VAD (2). According to WHO, VAD is of moderate to severe public-health importance in 122 countries (2). Globally, 190 million preschool children and 19 million pregnant women have low serum retinol concentration (2). In countries at risk of VAD, 33.3% of preschool children and 15.3% of pregnant women are deficient (2).

Several studies linked prenatal VAD with various adverse pregnancy and birth outcomes, including anaemia (5,6), preterm delivery (6-9), intra-uterine growth retardation (8,10), low birthweight (5,9,11), malformations (12), infection (13), pre-eclampsia/eclampsia (11,13-15), vertical transmission of HIV (16), poor infant growth (5,11), neonatal and infant mortality (5,17), and maternal mortality (17,18). Nevertheless, the negative effects of prenantal VAD on birth outcomes are still controversial.

Studies conducted over decades consistently indicated the public-health significance of VAD in Ethiopia (19). However, the full extent of the problem in pregnant women is not clearly known as most of the studies were carried on preschool children. Although WHO estimated 13.2% prevalence of VAD in pregnant women in Ethiopia (2), the available three studies (20-22) conducted in the southern and north-western part of the country reported higher prevalence figures ranging from 17 to 27%.

The objectives of the current study are to assess the prevalence and potential risk factors of prenatal VAD in Sidama zone, Southern Ethiopia, where an earlier survey (21) reported seriously high (27%) prevalence of VAD. The current study explored wide range of potential risk factors, including maternal literacy level, income, age, gestational age, household wealth index, agro-ecological zone, zinc deficiency (ZD), parity, birth interval, history of breastfeeding, level of C-reactive protein (CRP), dietary diversity (DD), type of staple food, distance from the nearby health facility, antenatal care (ANC), and history of nutrition education during pregnancy. A previous study (23) has already presented the prevalence and correlates of ZD among similar group of study subjects.

## MATERIALS AND METHODS

### Study design

This is a community-based, cross-sectional study, with descriptive and analytic designs.

### Setting

The study was conducted in January 2011 in 18 kebeles of Sidama zone, Southern Ethiopia. A kebele is the smallest administrative unit in Ethiopia, comprising approximately 1,000 households. Sidama zone is one of the 15 zones of Southern Nations Nationalities and Peoples Regional State (24). The zone has a population of 2,966,652 and population density of 430/km^2^ (24). In terms of agro-ecological zone, approximately 50%, 30%, and 20% of the people dwell in the midlands (1,750-2,300 m above sea-level (ASL)), highlands (>2,300 m ASL) and lowlands (<1750 m ASL) respectively (25). Livelihood of about 85% of the population depends on subsistent farming. Major crops grown in the area are enset (*Enset ventricosum*), coffee, and maize (25).

### Sample-size

Single proportion sample-size calculation formula was used in determining adequate sample-size for estimating the prevalence of VAD. The sample-size of 666 pregnant women was computed based at 95% confidence level, 5% margin of error, design effect of 2, 27% expected prevalence of VAD (21), and 10% non-response rate. However, to maximize the sample-size for the analytic component of the study, 750 pregnant women were recruited. The adequacy of the available sample-size for investigating the risk factors of VAD was assessed using the STATCALC application of Epi Info (version 3.5) at 95% confidence level, 80% power, and 1:1 ratio between deficient and non-deficient subjects.

### Sampling technique

Initially, all rural kebeles in the zone (total of 456 kebeles) were listed and stratified into three agro-ecological zones, namely lowlands (<1,750 m ASL), midlands (1,750-2,300 m ASL), and highlands (>2300 m ASL). Then the total sample-size (750 pregnant women) was divided into three strata (agro-ecological zones) proportionally to their population-size (20%, 50%, and 30% respectively). Six kebeles from every stratum—a total of 18 kebeles—were selected using simple random-sampling technique. Again, the sample-size allotted for each of the stratum was distributed among the respective six kebeles proportionally to their population-size.

In every selected kebele, house-to-house enumeration was conducted to identify pregnant women. Presumptive symptoms of pregnancy (ammenorrhea and/or increased uterine-size), with subsequent urine test for pregnancy (INSTANT-HCG ®, USA), were used for diagnosis of pregnancy. Eventually, 750 subjects were selected using systematic random-sampling technique.

### Data-collection method

A structured and pretested questionnaire was used for assessing potential correlates of VAD. The section of the questionnaire on socioeconomic information was adopted from standard DHS questionnaire, and the part on DD was taken from Food and Nutrition Technical Assistance (FANTA) indicator guideline (26). Other parts of the tool were developed by the principal investigators. The content validity of the tool was assessed against the conceptual framework of the study by relevant professionals, and the reliability of the tool was evaluated using test-retest method.

Data were collected by three trained clinical nurses in private setting within the compound of the nearby health posts. The questionnaire was administered using local language. The altitudes of the study area (in metre above sea-level) was measured at the centre of the kebeles, using Global Positioning System (GPS) (Magellan®, USA), and the kebeles were classified as highlands, midlands, and lowlands based on the aforementioned cutoff points.

Household dietary diversity score (DDS) quantifies DD, based on a 24-hour recall method. Respondents were asked whether they had taken any food from predefined 12 food categories in the previous day of the survey. Accordingly, the DDS was computed out of a score of 12. According to the recommendation of FAO (27), the DDS was categorized into low (DDS ≤3), medium (DDS of 4 or 5), or high (DDS ≥6).

Mid-upper arm-circumference (MUAC) of the women was measured to the nearest 0.1 cm, using MUAC tape. The measurement was taken on the middle left arm at relaxed position, without any clothing and with optimal tape tension. Severe acute malnutrition (SAM) was defined as MUAC less than 220 mm (28).

### Blood sample collection, serum extraction, and laboratory analysis

Venous blood was collected using stainless steel needles and plain tubes (SARSTEDT Monovette®, Germany). The blood was clotted for 20 minutes and consecutively centrifuged at 3000×g for 10 minutes. Visibly-haemolyzed samples were discarded. Serum was extracted and transferred immediately into screw-top vials. The samples were transported in icebox, protected from direct light, and kept frozen at −20 ºC until analyzed.

Serum retinol and zinc concentrations were determined at Ethiopian Health and Nutrition Research Institute following standard procedures, using high-performance liquid chromatography (Shimadzu®, Japan) and flame atomic absorption spectrometer (Varian SpectrAA®, Australia) respectively. VAD (serum retinol <0.7 µmol/L) and marginal VAD (serum retinol 0.7 to 1.05 µmol/L) were defined according to the recommendation of WHO (2) whereas ZD (serum zinc <8.6 µmol/L during the first trimester or <7.6 µmol/L during the second or third trimester) was defined according to the recommendation of International Zinc Nutrition Consultative Group (29). CRP was determined qualitatively, using Latex test (HumaTex CRP®, Germany). A distinctively visible agglutination was indicative of positive CRP result (CRP >5 mg/dL).

### Data analysis

Data-entry, screening, and analysis were carried out using SPSS 19.0 for Windows. The distribution of serum retinol level was assessed using Q-Q plot and found to be normal. Descriptive analysis was done using mean, frequency, and percentage. Independent *t*-test and one-way analysis of variance (ANOVA) were used in comparing retinol level across categories of independent variables. The assumptions of ANOVA (normal distribution and homoscedasticity of the dependent variable across the categories of independent variables) were checked to be fulfilled.

Wealth index was computed as a composite indicator of living standard, based on ownership of selected household assets, size of agricultural land, quantity of livestock, materials used for house construction, and ownership of improved water and sanitation facilities. The variables were reduced to a continuous variable, using principal component analysis. Ultimately, the five wealth index categories (poorest, poorer, middle, richer, and richest) were generated by splitting the continuous variable into 5 equal quintiles.

Logistic and linear regression analyses were used in controlling potential confounders. Independent variables significantly (p<0.05) associated with the dependent variable in simple regression models were exported to multiple regression models for adjustment. In multivariate logistic regression analyses, variables were entered separately into two models—distal and proximate. Factors which can directly affect serum retinol level were considered proximate factors whereas factors which have to function through the proximate factors to affect serum retinol level were considered distal factors. The distal model comprised agro-ecological zone, wealth index, maternal education, and women's participation in income-generating activities (IGAs). The proximate model included maternal age, gestational age, CRP level, DDS, and consumption of foods of animal source in the preceding day of the survey, ZD, distance from the nearby health facility, ANC, history of nutrition education during pregnancy, and SAM status. In the multivariate linear regression model, only the proximate factors were modelled as the *r*-squared value for distal model was extremely low.

The major assumptions of logistic regression analysis (absence of influential cases, multicollinearity and interaction among independent variables) and linear regression analysis (normally-distributed error terms, linear relation between dependent and independent variables, homoscedasticity, and absence of multicollinearity) were checked to be satisfied. The fitness of logistic and linear regression models were assessed using Hosmer-Lemeshow statistic and adjusted *r*-squared values respectively.

### Ethical considerations

The study was conducted in confirmation of the national and international ethical guidelines for biomedical research involving human subjects. Ethical clearance was granted by the institutional review board of Addis Ababa University. Informed written consent was taken from the study subjects. Needle safety procedures were in line with international standards. After data collection, the data collectors provided nutrition education to all study participants.

## RESULTS

### Background information on study subjects

Of 750 pregnant women sampled, 700 (93.3%) were willing to take part in the study. Nearly half of the study participants—357 (51.0%)—were from the midlands whereas the remaining 187 (26.7%) and 156 (22.3%) were from highlands and lowlands respectively.

The mean age (±standard deviation) of the respondents was 28.5±5.5 years. About 50 (7.2%), 347 (49.6%), and 303 (43.3%) were in the first, second and third gestational trimester respectively. Nearly two-thirds—462 (66.0%)—had no education, and 561 (80.1%) were housewives. The vast majority—679 (97.0%) and 596 (85.1%)—were *Sidama* in ethnicity and Protestants in religion respectively. The average household-size was 4.3±1.8. The average size of agricultural land per household was 0.38±0.19 hectares.

### Prevalence of vitamin A deficiency

The mean serum retinol level was 0.84±0.41 µmol/L (95% CI 0.81-0.87). The levels in the first, second and third trimester were 0.97±0.40, 0.89±0.42 and 0.76±0.40 µmol/L respectively. The retinol level in the third trimester was significantly lower than the corresponding values for the first and second trimesters (F=11.08, p=0.000). Based on the cutoff point recommended by WHO (2), 265 [37.9% (95% CI 34.3-41.5)] of the subjects had VAD, and additional 201 [28.7% (95% CI 25.3-32.1)] subjects had marginal VA status (Figure). Pregnant women in the third trimester had 2.59 (95% CI 1.23-5.48) times increased odds of VAD compared to those in the first trimester.

**Figure.**
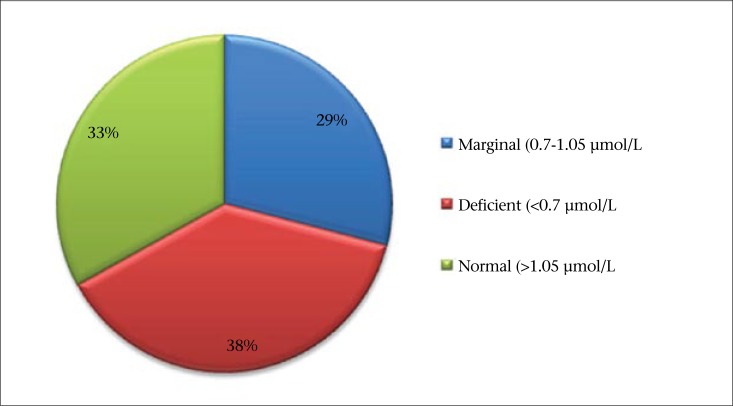
Prevalence of vitamin A deficiency among pregnant women in rural Sidama, Southern Ethiopia, February 2011

About 59 (8.4%) of the blood samples were positive for CRP (CRP ≥5 mg/dL). The retinol levels for CRP-negative and positive samples were 0.85±0.42 and 0.66±0.34 µmol/L respectively. The difference was significant (*t*=3.828, p=0.000). Elevated CRP was associated with 22.5% reduction in serum retinol concentration. Positive CRP result was significantly associated with VAD with AOR 2.21 (95% CI 1.22-4.02).

### Correlates of vitamin A deficiency

#### Sociodemographic factors

Maternal age was negatively associated with VAD. The retinol concentrations for women aged 15-24, 25-34, and 35-49 years were 0.89±0.44, 0.85±0.40, and 0.71±0.41 µmol/L respectively. The global difference was significant (F=6.890, p=0.001). The odds ratio of VAD was 2.23 (95% CI 1.31-3.81) times higher in the oldest participants compared to the youngest group.

The study witnessed no significant association between household wealth index and maternal VA status. Nevertheless, women's participation in IGAs was positively associated. Retinol level among women who were involved in IGA was significantly higher than their counterparts (*t*=2.557, p=0.012). Likewise, women who were devoid of self-income had 2.18 (95% CI 1.29-3.69) times higher odds of VAD.

Maternal education showed a positive influence on VA status. The retinol levels among the women who had no education, who completed first to fourth grade and beyond fourth grade were 0.80±0.43, 0.87±0.38, and 0.93±0.38 µmol/L respectively. The global difference across the categories was significant (F=19.682, p=0.000). Women without education had 1.73 times (95% CI 1.02-2.92) higher odds of VAD compared to women educated beyond the fourth grade.

The odds of VAD were not significantly different across the three agro-ecological zones.

#### Nutritional factors

Dietary diversity level was assessed, based on a 24-hour recall method. The diet was predominated by roots and tubers, cereals, and legumes. In the reference period, 549 (78.4%), 369 (52.7%), and 366 (52.2%) consumed roots and tubers (mainly enset), cereals (mainly maize), and legumes (mainly broad bean and kidney bean) respectively. Only 199 (28.4%) and 181 (25.6%) reported consumption of provitamin A and preformed vitamin A-rich foods respectively.

Based on ANOVA and logistic regression analyses, the level of DD and consumption of foods from animal source in the reference period were positively associated with VA status. Women with low DDS had 1.94 (95% CI 1.17-3.19) times increased odds of VAD compared to women with high DDS. Those who did not consume foods from animal source in the preceding day had 1.51 times (95% CI 1.04-2.13) higher odds than their counterparts.

The retinol level among respondents who considered maize as their staple diet (0.88±0.42 µmol/L) was significantly higher than those who reported enset [(0.81±0.41 µmol/L) (*t*=2.028, p=0.043)]. Nonetheless, the association was not significant in the logistic model. Similarly, VA status was not significantly associated with SAM status (Table 1).

VAD and ZD tend to occur together. A quarter—24.9% (95% CI 21.7-28.1)—of the subjects had both of the deficiencies, and about two-thirds—65.6% (95% CI 59.9-71.4)—of the vitamin A-deficient subjects were zinc-deficient. After controlling for joint nutritional and non-nutritional determinants, VAD and ZD were significantly associated with AOR of 1.80 (95% CI 1.28-2.53).

#### Reproductive and healthcare factors

Parity and VA status were negatively associated. Significant difference in retinol concentrations was witnessed across parity of 0, 1-2, 3-4, and 5 or more (F=2.886, p=0.035). Compared to nulliparas, grand multiparas had 1.92 times (95% CI 1.02-3.64) higher odds of VAD.

Among 553 subjects who gave at least one birth in the preceding five years of the survey, the interval between the previous birth and the expected date of birth in the index pregnancy was estimated. The retinol levels in women with short (<24 months) and optimal (≥24 months) birth intervals were 0.72±0.45 and 0.84±0.41 µmol/L respectively. The difference was significant (*t*=2.032, p=0.043). Women with short birth interval had 2.11 times (95% CI 1.15-3.86) higher odds of VAD.

The association between duration of breastfeeding and VA status was assessed among 492 women who reported history of breastfeeding in the previous five years of the survey. The retinol concentrations in mothers who breastfed for durations less than 18 months, 18-24 months and greater than 24 months, were 0.99±0.42, 0.87±0.42, and 0.79±0.40 µmol/L respectively. The global difference was significant (F=6.491, p=0.002). Post-hoc Tukey test identified that the retinol level among women who breastfed for more than 24 months was significantly lower than those who breastfed for less than 18 months.

Serum retinol level and odds of VAD were compared across various healthcare service-related factors. However, physical distance from the nearby health facility, frequency of ANC, and receiving nutrition education during pregnancy were not associated with VA status (Table 1).

**Table 1.** Association between vitamin A status and various potential risk factors among pregnant women in rural Sidama, Southern Ethiopia, February 2011

Variable	Serum retinol µmol/L (mean ±SD)	VAD+	VAD-	Crude OR (95% CI)	Adjusted OR (95% CI) **
Staple diet					
Enset-based	0.81±0.41	180	271	1.28 (0.93-1.77)	-
Cereal-based	0.88±0.42	85	164	1r	-
Dietary diversity in the previous day					
Low (DDS ≤3)	0.75±0.41	119	124	2.55 (1.63-4.00)*	1.94 (1.17-3.19)*
Optimal (DDS of 4 or 5)	0.87±0.41	108	210	1.37 (0.88-2.12)	1.10 (0.70-1.72)
High (DDS ≥6)	0.91±0.43	38	101	1^r^	1^r^
Consumed foods from animal source in the previous day					
No	0.81±0.42	216	303	1.92 (1.33-2.78)*	1.51 (1.04-2.13)*
Yes	0.91±0.39	49	132	1^r^	1^r^
Severe acute malnutrition					
No (MUAC ≥220 mm)	0.87±0.42	178	330	1^r^	1^r^
Yes (MUAC <220 mm)	0.75±0.39	87	105	1.54 (1.10-2.15)*	1.26 (0.88-1.81)
Zinc deficiency status					
Non-deficient	0.92±0.41	91	238	1^r^	1^r^
Deficient	0.76±0.40	174	197	2.31 (1.68-3.17)*	1.80 (1.28-2.53)*
Parity					
0	0.89±0.40	34	82	1^r^	1^r^
1-2	0.85±0.41	102	174	1.41 (0.86-2.26)	1.14 (0.69-1.87)
3-4	0.84±0.42	91	140	1.57 (0.97-2.53)	1.35 (0.81-2.26)
≥5	0.72±0.40	38	39	2.35 (1.29-4.28)*	1.92 (1.02-3.64)*
Walking distance from nearby health facility					
0-30 minutes	0.83±0.41	229	369	1^r^	-
Longer than 30 minutes	0.89±0.44	36	66	0.88 (0.57-1.36)	-
Number of ANC follow-ups					
0	0.87±0.41	121	243	1^r^	1^r^
1-2	0.80±0.43	134	173	1.56 (1.14-2.13)*	1.42 (0.97-2.02)
≥3	0.86±0.41	10	19	1.06 (0.48-2.34)	0.70 (0.30-1.64)
Received nutrition education during pregnancy					
Yes	0.80±0.41	70	105	1^r^	-
No	0.85±0.42	195	330	0.89 (0.62-1.26)	-

*Statistically significant association at 0.05 level of significance;

**Adjusted for all significant variables in the table plus gestational age and CRP level; CI=Confidence interval; r=Reference value; SD=Standard deviation

### Linear regression modelling

In the linear regression model, about 26.2% of the variability in serum retinol level (μmol/L) was explained by 8 independent variables (Table 2). Unit increment in maternal age (in years), parity, and gestational age (in months) were associated with 0.01, 0.04, and 0.03 μmol/L decline in serum retinol level respectively. On the contrary, a unit increment in DDS and MUAC (in cm) was associated with 0.04 and 0.02 μmol/L rise in retinol concentration. Serum zinc and retinol concentrations were positively associated with unstandardized coefficient of 0.03. An adjusted net difference of 0.12 μmol/L was observed between CRP-positive and negative samples. Similarly, a significant difference of 0.12 μmol/L was witnessed between those who consumed foods from animal source in the previous day of the survey and their counterparts.

**Table 2.** Linear regression output on the predictors of serum retinol concentrations (μmol/L) among pregnant women in rural Sidama, Southern Ethiopia February 2011

Independent variable	Unstandardized coefficient	*t*-statistic	p value
Constant	0.481		
Maternal age (years)	-0.008	-2.474	0.014
Parity (0-9)	-0.036	-3.343	0.001
Gestational age (months)	-0.030	-3.730	0.000
Diet diversity level in the previous day (0-12)	0.040	4.429	0.000
Mid-upper arm-circumference (cm)	0.018	2.515	0.012
Consumed foods from animal source in the previous day (0=no, 1=yes)	0.117	3.575	0.000
CRP (0=Negative, 1=Positive)	-0.120	-2.339	0.020
Serum zinc concentration (in μmol/L)	0.034	3.545	0.000

## DISCUSSION

According to WHO, the public-health significance of VAD is considered to be of severe degree when the prevalence of low serum retinol (<0.70 μmol/L) in pregnant women exceeds 20% from the reference value (2). Accordingly, with the prevalence of 37.9%, VAD is of severe public-health importance in the area.

As the study is cross-sectional, the reported prevalence is liable to seasonality bias. If the study had been conducted in drought-prone months, like May, June, and July, a higher prevalence of VAD would have been anticipated. On the other hand, the study might have over-estimated the prevalence as women in the first trimester were largely under-represented.

Few studies attempted to determine the prevalence of subclinical VAD among pregnant women in Ethiopia. Gibson *et al*. reported 27% prevalence of VAD among 85 pregnant women in three kebeles of Sidama (21). The prevalence is comparable with the current study in the sense that the confidence intervals overlapped. However, the apparent difference in the point estimators might be due to the fact that the earlier study included conveniently-selected women in relatively accessible kebeles.

Two studies conducted among ANC attendants in Gondar Hospital, Northwestern Ethiopia, found 17.2% (20) and 18.4% (22) prevalence of subclinical VAD. The current study reported relatively higher prevalence, probably due to the reason that the earlier studies were conducted in an urban setup where prevalence of VAD is expected to be lower. According to EDHS 2005, the prevalence of maternal night-blindness in rural areas of Ethiopia was two times higher than that in the urban areas (30).

Gestational trimester and VA status were negatively associated, apparently due to the effect of haemodilution. Parallel findings were reported in Zimbabwe (31), India (32), and Turkey (33). In the current study, serum retinol significantly declined by about 22% from the first (0.97 µmol/L) to the third trimester (0.76 µmol/L). A study in Indonesia also reported a comparable 20% reduction (34). The finding suggests the need for having trimester-specific cutoff points for defining prenatal VAD.

Elevated CRP was associated with 22.5% reduction in serum retinol concentration. The finding is consistent with the understanding that inflammation causes reduction in serum retinol as a consequence of the acute-phase response (1,35). A meta-analysis also concluded that elevated CRP protein is associated with 25% decline in retinol level (36). However, in the current study, the negative association observed between CRP and retinol levels can also be the manifestation of vicious causal relationship between infection and VAD.

Analysis based on the data of the third US National Health and Nutrition Examination Surveys concluded that serum retinol among females remains constant between 20 and 49 years of age (37). However, in the current study, maternal age and VA status were negatively associated. The reason for divergence might be: in the developing world, repeated nutritionally-deleterious lifetime events, like extended breastfeeding and drought, frequently occur and deplete maternal store of retinol across the lifespan. Ethiopian DHS 2005 also found that the prevalence of maternal night-blindness tends to increase with age (30). Studies in India (38) and Nepal (39) concluded likewise.

Unlike many previous findings (38-40), household wealth status was not associated with VAD status. The unexpected finding might be explained based on the reason that household wealth status was measured using a relative rather than an absolute index. It means, as the study population had minimal wealth variation, wealth index might have lacked the ability to detect significant association with VAD. Nevertheless, involvement of women in IGAs was found to have beneficial effect, irrespective of household wealth and maternal education status.

History of too close and too many births was associated with higher odds of VAD. This is consistent with the understanding that repeated reproductive cycles deplete maternal store of nutrients (41). Previous studies in the developing world also reported the same. A study in Southern India reported a twofold increased risk of night-blindness among grand multiparas compared to nulliparas women (38). Studies in Cambodia (40) and Nepal (39) found 1.5 and 1.6 times higher risk of night-blindness respectively in women with 3 or more previous births compared to their counterparts.

VAD and ZD were positively associated in our study. Potential nutritional and non-nutritional confounders cannot explain the association as they had been statistically controlled. The association can be secondary to the influence of zinc on the metabolism of vitamin A (42). A study in pregnant rhesus monkeys concluded that, in the zinc-deficient groups, zinc affects vitamin A metabolism by altering the formation or release of holo-retinol-binding protein (43). Zinc supplementation trial in pregnant Nepalese women also suggested that zinc potentiates the effect of vitamin A in restoring night-vision among night-blind pregnant women with low initial serum zinc concentrations (44).

Distance from the nearby health facility, prenatal nutrition education/counselling, and frequency of ANC were not associated with vitamin A status. This might be due to the reason that nutritional care is not well-integrated into maternity services. In addition, the provision of nutrition education might not be effective in the absence of concurrent livelihood promotion strategies.

### Conclusions

The study was conducted in socioeconomically-disadvantaged and food-insecure locality. With a prevalence of 38%, VAD has severe degree of public-health significance in the area. Advanced gestational age was negatively associated with serum retinol level, indicating that there is a need for developing trimester-specific cutoff points for defining prenatal VAD. Elevated CRP, advanced age, inferior maternal socioeconomic status, dependence on poorly-diversified and plant source-based diets, zinc deficiency, and history of too close and too many births were pertinent risk factors of VAD. ANC and nutrition education were not potent enough in reducing the burden of VAD. VAD should be combated through improving diet diversity and expansion of family-planning services in the area. Socioeconomic empowerment of women would also have positive contribution. Nutritional care should be integrated into maternity services.

## ACKNOWLEDGEMENTS

Special thanks go to all study subjects who volunteered to participate in the study. The authors acknowledge Addis Ababa University School of Public Health for funding the study and Ethiopian Health and Nutrition Research Institute for conducting the laboratory analyses.
